# Catheter ablation vs. anti-arrhythmic drug therapy for ventricular tachycardia in ischaemic heart disease: a meta-analysis of randomized controlled trials

**DOI:** 10.1093/europace/euaf302

**Published:** 2025-11-29

**Authors:** Francesco Santoro, Giacomo Mugnai, Laura Perrotta, Boldizsar Kovacs, Leon Dinshaw, Alvaro Marco del Castillo, Christiane Jungen, Stefan Kurath-Koller, Stefan Stojković, Bert Vandenberk, Kevin Vernooy

**Affiliations:** Cardiology Unit, Department of Medical and Surgery Sciences, University of Foggia, Viale Pinto n.1, 71122 Foggia, Italy; Department of Cardiology, Cardiovascular Research Institute Maastricht (CARIM), Maastricht University Medical Center +, Maastricht, The Netherlands; Department of Cardiology, Cardiovascular Research Institute Maastricht (CARIM), Maastricht University Medical Center +, Maastricht, The Netherlands; Division of Cardiology, Cardio-Thoracic Department, University Hospital of Verona, Verona, Italy; Department of Cardiology, Cardiovascular Research Institute Maastricht (CARIM), Maastricht University Medical Center +, Maastricht, The Netherlands; Arrhythmia Unit, Department of Cardiology, Careggi University Hospital, Florence, Italy; Department of Cardiology, Cardiovascular Research Institute Maastricht (CARIM), Maastricht University Medical Center +, Maastricht, The Netherlands; Department of Cardiology, University Hospital Bern, Bern, Switzerland; Department of Cardiology, Cardiovascular Research Institute Maastricht (CARIM), Maastricht University Medical Center +, Maastricht, The Netherlands; Department of Cardiology, Sana Hanse Klinikum, Wismar, Germany; Department of Cardiology, Cardiovascular Research Institute Maastricht (CARIM), Maastricht University Medical Center +, Maastricht, The Netherlands; Department of Cardiology, Hospital Universitario 12 de Octubre, Madrid, Spain; Department of Cardiology, Cardiovascular Research Institute Maastricht (CARIM), Maastricht University Medical Center +, Maastricht, The Netherlands; West German Heart and Vascular Center, Department of Cardiology and Vascular Medicine, University Hospital Essen, Essen, Germany; Department of Cardiology, Cardiovascular Research Institute Maastricht (CARIM), Maastricht University Medical Center +, Maastricht, The Netherlands; Division of Pediatric Cardiology, Department of Pediatrics, Medical University of Graz, Graz, Austria; Department of Cardiology, Cardiovascular Research Institute Maastricht (CARIM), Maastricht University Medical Center +, Maastricht, The Netherlands; Department of Internal Medicine II, Division of Cardiology, Medical University of Vienna, Vienna, Austria; Department of Cardiology, Cardiovascular Research Institute Maastricht (CARIM), Maastricht University Medical Center +, Maastricht, The Netherlands; Department of Cardiovascular Sciences, KU Leuven, Leuven, Belgium; Department of Cardiology, Cardiovascular Research Institute Maastricht (CARIM), Maastricht University Medical Center +, Maastricht, The Netherlands

**Keywords:** Ventricular tachycardia, Catheter ablation, Anti-arrhythmic drugs, Amiodarone, Sotalol, Implantable cardioverter-defibrillator, Ischaemic cardiomyopathy, Re-hospitalization

## Abstract

**Aims:**

Ventricular tachycardia (VT) in ischaemic heart disease (IHD) requires complex management strategies including catheter ablation (CA) and anti-arrhythmic drugs (AADs). The aim of this study is to compare efficacy and safety of CA vs. AADs in patients with IHD and VT.

**Methods and results:**

We performed a meta-analysis of randomized controlled trials (RCTs) enrolling patients with IHD and ICD randomized to CA or AADs. Primary outcome was appropriate ICD therapy. Secondary outcomes included inappropriate ICD therapy, cardiovascular (CV) re-hospitalization, all-cause/CV mortality, and adverse events. Subgroup analyses were conducted for amiodarone and sotalol, with an exploratory evaluation of a composite endpoint (ICD shock, VT storm, all-cause death). Four RCTs including 947 patients (mean age 68 ± 2 years; 93% male) were analysed. CA significantly reduced the risk of appropriate ICD therapy compared with AADs (149/470 [31.7%] vs. 229/477 [48.0%]; RR 0.81; 95% CI [0.67, 0.97]; *P* = 0.02). Among secondary outcomes, CA decreased the incidence of CV re-hospitalization [RR 0.84; 95% CI (0.72, 0.99); *P* = 0.04] and adverse events [RR 0.42; 95% CI (0.28, 0.62); *P* < 0.01], while no differences were observed in all-cause/CV mortality and inappropriate ICD therapy. In subgroup analyses, CA was superior to sotalol in reducing the composite endpoint of ICD shock, VT storm and all-cause death [RR: 0.82, 95% CI (0.69, 0.98), *P* = 0.03]; whereas, no significant benefit was seen compared to amiodarone [RR: 0.92; 95% CI (0.78, 1.09), *P* = 0.32].

**Conclusion:**

In ischaemic heart disease and VT, CA compared with anti-arrhythmic drugs is associated with a reduction of appropriate ICD therapy, cardiovascular re-hospitalization, and adverse events with benefits most evident versus sotalol.

## Graphical Abstract

**Graphical Abstract:**
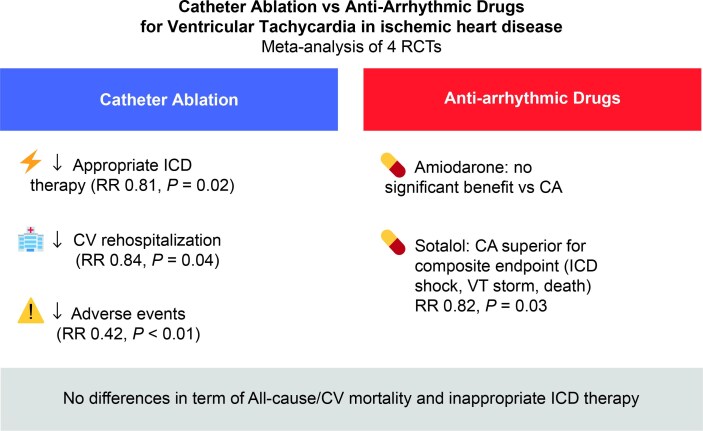

What's new?

- First meta-analysis restricted to anti-arrhythmics drugs vs. catheter ablation for ventricular tachycardia in ischaemic cardiomyopathy with additional sub-analysis on amiodarone and sotalol.
- Catheter ablation reduced ICD therapies, cardiovascular re-hospitalizations, and treatment-related complications compared with drug therapy.
- Outcomes were particularly favourable vs. sotalol (lower risk of composite endpoint ICD shock, VT storm, and death), while no clear benefit emerged over amiodarone

## Introduction

Ventricular tachycardia (VT) is a major cardiac adverse event that can complicate acute and chronic myocardial ischaemia.^1,2^ In the long-term, one out of five patients with ischaemic heart disease (IHD) develops myocardial scarring, usually detected through cardiac magnetic resonance imaging.^3–5^ The presence of myocardial scar or fibrosis pre-disposes to re-entrant circuits, which may result in sustained VT and has been associated with an increased risk of sudden cardiac death (SCD).^6^

Although implantable cardioverter-defibrillators (ICDs) may prevent SCD,^7^ they do not avoid VT occurrence or recurrences and can be associated with several complications, such as inappropriate ICD shocks^8^ and device-related infections.^9^ Moreover, appropriate ICD shocks have been associated with higher mortality rates across all ages.^10^ Anti-arrhythmic drugs (AADs), particularly amiodarone and sotalol, are commonly used for VT treatment but are limited by possible long-term toxicity.^11,12^

Catheter ablation (CA) targets the arrhythmogenic tissue and can reduce the VT burden in IHD.^13–15^ Several studies evaluated the timing of VT ablation, showing its efficacy before ICD implantation in terms of arrhythmia burden reduction^16^ but not for mortality or heart failure hospitalization.^17^ However, data from randomized controlled trials (RCTs) comparing CA and AADs, specifically in patients with IHD, are limited. We conducted a meta-analysis of all available RCTs enrolling only patients with IHD to compare the efficacy and safety of CA vs. AADs therapy. Additionally, an exploratory analysis of efficacy of amiodarone or sotalol vs. CA is provided.

## Methods

### Search strategy and study selection

This study was conducted in accordance with the PRISMA (preferred reporting items for systematic reviews and meta-analyses) guidelines. PubMed/MEDLINE, Embase, The Cochrane Central Register of Controlled Trials, and Scopus were systematically searched from database inception to July 2025.The meta-analysis also complied with the AMSTAR 2 (a measurement tool to assess systematic reviews) checklist to ensure methodological rigor and transparent reporting (see Supplementary material online, *Table S1*). The following keywords and MeSH terms were used in various combinations: ‘*ventricular tachycardia*’, ‘*VT*’, ‘*ischaemic cardiomyopathy*’, ‘*ischaemic heart disease*’, ‘*myocardial infarction*’, ‘*catheter ablation*’, ‘*anti-arrhythmic drugs*’, ‘*amiodarone*’, and ‘*sotalol*’. Reference lists of relevant articles were hand-searched to identify additional eligible studies.

Eligible studies were RCTs comparing CA with AADs therapy in patients with IHD and VT. Studies including non-ischaemic cardiomyopathy patients were excluded unless data for the ischaemic subgroup were separately reported.

**Figure 1:**
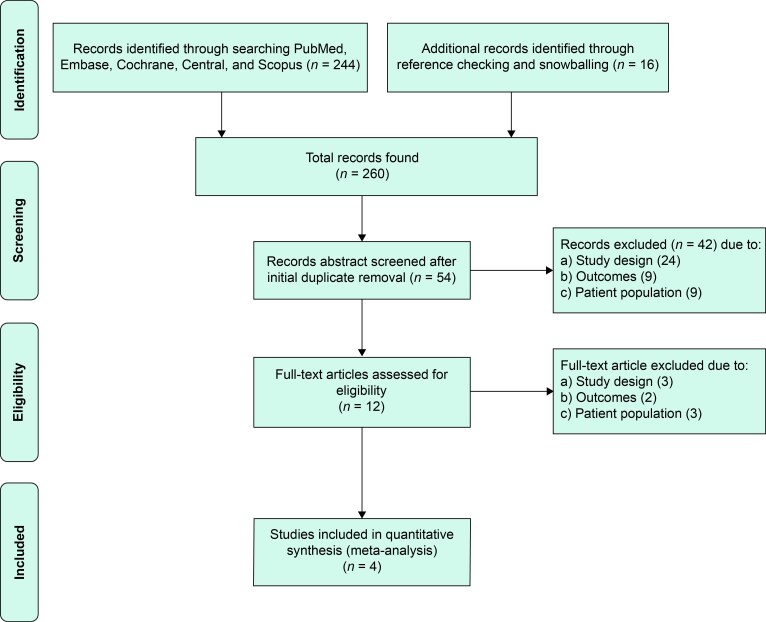
PRISMA flow diagram of study selection.

A total of 260 records were identified through database searching and reference checking. After duplicate removal and abstract screening, 12 full-text articles were assessed for eligibility. Of these, eight were excluded due to study design, outcomes, or patient population, leaving four RCTs that met inclusion criteria and were included in the meta-analysis (*Figure 1*).

### Data extraction and outcomes

Two independent reviewers (F.S. and G.M.) screened titles, abstracts, and full texts. Discrepancies were resolved by consensus with a third reviewer (L.P.). For each eligible trial, we extracted baseline demographics, sample size, intervention strategy (CA vs. AAD), follow-up duration, and reported outcomes.

Mean follow-up of the RCTs included was 31.5 ± 13.6 months.

The primary outcome was appropriate ICD therapy defined as device-delivered treatment (either anti-tachycardia pacing or shock) in response to VT or ventricular fibrillation. Secondary outcomes were inappropriate ICD shock therapy, cardiovascular (CV) re-hospitalization, all-cause and CV mortality, and adverse events. Subgroup analyses compared CA with amiodarone and with sotalol, with an exploratory evaluation of a composite endpoint of appropriate ICD shock, VT storm, and all-cause mortality.

Adverse events were defined as treatment-related complications. For CA, this included periprocedural complications, such as cardiac tamponade, stroke, vascular access complications, or procedure-related death. For AADs therapy, adverse events included drug-related toxicity, such as pulmonary or thyroid dysfunction, hepatotoxicity, tremor/ataxia, pro-arrhythmia, and severe bradyarrhythmia requiring intervention.

### Risk of bias and study quality assessment

The methodological quality of the included RCTs was assessed using the Cochrane Risk of Bias 2 (ROB 2) tool. Five domains were evaluated: randomization process, deviations from intended interventions, missing outcome data, measurement of outcomes, and selection of the reported result. Each domain was rated as *low risk*, *some concerns*, or *high risk*, and an overall judgment was derived for each study. Two reviewers performed the assessment independently, resolving discrepancies by consensus. Results of the quality appraisal are summarized in Supplementary material online, *Table S2*.

### Statistical analysis

Continuous variables were expressed as mean ± standard deviation. Pooled effect estimates were expressed as risk ratios (RRs) with 95% confidence intervals (CIs). A random-effects model (DerSimonian and Laird method) was applied, considering the expected clinical heterogeneity among trials. Statistical heterogeneity was quantified using the *I*² statistic and Cochrane *Q* test, with *I*² > 50% indicating moderate-to-high heterogeneity. Publication bias was explored using funnel plot asymmetry. All analyses were performed using Review Manager (RevMan, version 5.4.1). A two-tailed *P*-value <0.05 was considered statistically significant.

## Results

### Patient population

Four RCTs were selected and a total of 947 patients were included in the analysis.^18–21^ Four hundred and seventy patients were allocated to the ablation group and 477 to the AADs group. Mean age was 68 ± 2 years and most of the patients were male (890 out of 947, 93%) (*Tables 1 and 2*).

**Table 1:** Design and methodological features of included randomized trials

Trial	First author (year)	*n* (total)	Design/intervention	Comparator	Follow-up (months)	Primary endpoint
SMASH-VT^18^	Reddy (2007)	128	ICD + prophylactic CA	ICD + AAD	22.5 ± 5.5	Survival free from appropriate ICD therapy
VANISH^19^	Sapp (2016)	259	CA + baseline AAD	Escalated AAD (↑amiodarone ± mexiletine)	27.9 ± 17.1	Composite: death, VT storm, or appropriate ICD shock
SURVIVE**-**VT^20^	Arenal (2022)	144	Substrate-based CA	AAD (amiodarone ± β-blocker or sotalol ± β-blocker)	24 (median)	Composite: CV death, ICD shock, HF hospitalization, or severe AE
VANISH-2^21^	Sapp (2025)	416	Substrate-based CA	AAD (sotalol or amiodarone)	4.3 years (median)	Composite: death, VT storm, ICD shock, or sustained VT intervention

CA, catheter ablation; AAD, anti-arrhythmic drug; ICD, implantable cardioverter-defibrillator; AE, adverse event; CV, cardiovascular; HF, heart failure.

**Table 2:** Baseline clinical and echocardiographic characteristics of patients

Trial	Mean age (years)	Male (%)	LVEF (%)	NYHA II–III (%)	Prior MI (%)	Diabetes (%)	HTN (%)	Prior ICD (%)	VT storm (%)
SMASH-VT^18^	67 ± 9	87	31 ± 9	62	100	24	68	100	18
VANISH^19^	68 ± 8	93	31 ± 10	69	100	29	66	100	13
SURVIVE-VT^20^	67 ± 9	96	34 ± 8	72	100	26	65	100	19
VANISH-2^21^	68 ± 10	94	34 ± 10	70	100	25	64	100	15

CA, catheter ablation; HTN, hypertension; ICD, implantable cardioverter-defibrillator; LVEF, left ventricular ejection fraction; NYHA, New York Heart Association; MI, myocardial infarction; VT, Ventricular Tachycardia.

### Primary outcome

**Figure 2:**
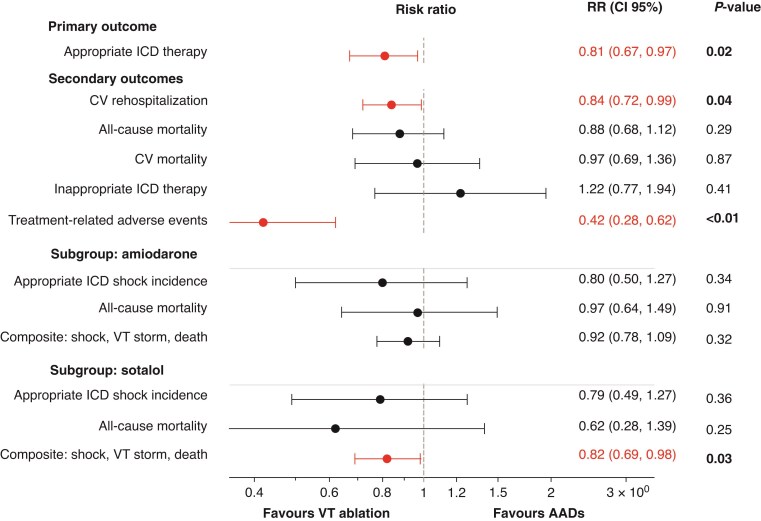
Forest plot illustrating the RR of appropriate ICD therapy, CV re-hospitalization, all cause/CV mortality, inappropriate ICD therapy and adverse events among patients treated with VT ablation or AADs with sub-analysis including patients treated with amiodaron and sotalol. AADs, anti-arrhythmic drugs; CV, cardiovascular; ICD, implantable cardioverter-defibrillator; VT, ventricular tachycardia.

CA was associated with a significantly lower incidence of appropriate ICD therapy compared to AADs during a mean follow-up of 31.5 ± 13.6 months [RR 0.81; 95% CI (0.67, 0.97); *P* = 0.02, *I*² = 61%] (*Figure 2*; Supplementary material online, *Figure S1* and *S2*).

### Secondary outcomes

CA reduced the risk of CV re-hospitalization [RR 0.84; 95% CI (0.72, 0.99); *P* = 0.04, *I*² = 56%, Supplementary material online, *Figure S1*]. In contrast, no differences were detected for all-cause mortality [RR 0.88; 95% CI (0.68, 1.12); *P* = 0.29, *I*² = 0%, Supplementary material online, Figure  *S3*], CV mortality [RR 0.97 (0.69, 1.36); *P* = 0.87, *I*² = 0%, Supplementary material online, Figure  *S3*] and inappropriate ICD therapy [RR 1.22; 95% CI (0.77, 1.94); *P* = 0.41, *I*² = 0%, Supplementary material online, *Figure S3*].

### Adverse events

Across trials, CA was associated with a significantly lower rate of treatment-related adverse events compared with AADs. Pooled analysis confirmed a lower incidence of adverse events with ablation [RR 0.42; 95% CI (0.28, 0.62); *I*² = 18%, *P* < 0.01] (*Figure 2* and *Table 3*; Supplementary material online, *Figure S1*).

**Table 3:** Adverse events in ventricular tachycardia ablation vs. anti-arrhythmic drugs

Trial	Ablation group (*n*, %)	AAD group (*n*, %)
VANISH^19^	Total = 8/132 (6%)	Total = 27/127 (21.2%)
	Cardiac perforations (*n* = 2); major bleeding (*n* = 3); vascular injury (*n* = 3)	Deaths *n* = 3 (pulmonary toxicity *n* = 2; hepatic failure *n* = 1)
		Pulmonary infiltrate (*n* = 2)
		Hyperthyroidism (*n* = 5)
		Hypothyroidism (*n* = 5)
		Hepatic Dysfunction (*n* = 6)
		Tremor/Ataxia (*n* = 6)
SURVIVE**-**VT^20^	Total = 6/71 (8.4%)	Total 8/73 (10.9%)
	Severe pericardial effusion (*n* = 1)	Hyperthyroidism (*n* = 1)
		Hypothyroidism (*n* = 1)
	Acute pulmonary oedema (*n* = 1)	Pulmonary toxicity (*n* = 2)
		Significant bradycardia and/or intolerance (*n* = 4)
	Cardiogenic shock (*n* = 1)	
	Pseudoaneurysm requiring surgery (*n* = 1)	
	Stroke/TIA (*n* = 2)	
VANISH2^21^	Total = 17/203 (7.9%)	Total = 41/213 (19.2%)
	Deaths (stroke *n* = 1, pneumonia *n* = 1)	Tremor/ataxia (*n* = 3)
		Pulmonary infiltrate/fibrosis (*n* = 5)
	Vascular injuries with pseudoaneurysm (*n* = 5 including 2 pts with major Bleeding)	Symptomatic bradycardia (*n* = 2)
		Hepatic dysfunction (*n* = 8)
		Heart block (*n* = 1)
		Hyperthyroidism (*n* = 13)
		Hypothyroidism (*n* = 9)
	Stroke (*n* = 3)	
	Cardiac perforation (*n* = 1)	
	Peripheral embolism (*n* = 1)	
	Persistent heart block (*n* = 2)	
	VF arrest (*n* = 1), subdural hematoma (*n* = 1), and bladder mechanical trauma (*n* = 1)	

### Subgroup analyses

#### CA vs. amiodarone therapy

Three randomized trials were selected for the comparison between CA and amiodarone therapy. A total of 612 patients were included in this analysis. Three hundred and fifty-nine patients were allocated to the ablation group and 253 to the Amiodarone group. There were no differences between CA and amiodarone therapy in terms of appropriate ICD shock incidence [RR: 0.80, 95% CI (0.50, 1.27), *I*² = 75%, *P* = 0.34], all-cause mortality [RR: 0.97 (0.64, 1.49), *I*² = 0%, *P* = 0.91], and the composite endpoint of appropriate ICD Shock, VT storm and all-cause mortality [RR: 0.92; 95% CI (0.78, 1.09), *I*² = 57%, *P* = 0.32] (*Figure 2*; Supplementary material online, *Figure S4*). However, CA markedly reduced treatment-related adverse events compared with amiodarone (RR 0.25; 95% CI 0.10–0.60; *I*² = 18%, *P* = 0.01).

#### CA vs. sotalol therapy

Three randomized trials were selected for the comparison of CA with sotalol therapy. A total of 477 patients were included in the analysis. Three hundred and twenty-one patients were allocated to the ablation group and 156 to the Sotalol group. There were no differences between CA and Sotalol therapy in terms of appropriate ICD shock incidence [RR: 0.79, 95% CI (0.49, 1.27), *I*² = 68%, *P* = 0.36] and all-cause mortality [RR: 0.62, 95% CI (0.28, 1.39), *I*² = 0%, *P* = 0.25]. However, CA was associated with a lower incidence of the composite endpoint of appropriate ICD shock, VT storm, and all-cause mortality [RR: 0.82, 95% CI (0.69, 0.98), *I*² = 0%, *P* = 0.03] and a trend towards fewer treatment-related adverse events (RR 0.55; 95% CI 0.25–1.10; *I*² = 18%, *P* = 0.11) (*Figure 2*; Supplementary material online, *Figure S4*).

### Risk of bias assessment

All four randomized trials demonstrated low risk of bias for the randomization process and outcome measurement. No study was judged at high overall risk of bias. A detailed domain-by-domain evaluation is provided in Supplementary material online, *Table S2*.

## Discussion

**Figure 3:**
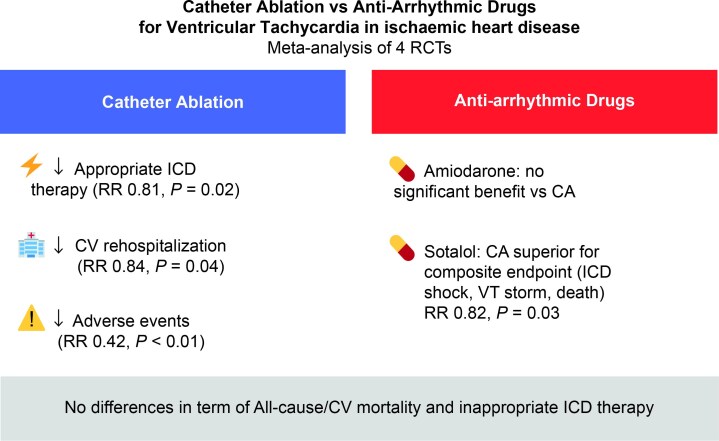
CA vs. anti-arrhythmic drugs for ventricular tachycardia in ischaemic heart disease: meta-analysis of randomized controlled trials. CA, catheter ablation; CV, cardiovascular; ICD, implantable cardioverter-defibrillator; VT, ventricular tachycardia.

We report an updated meta-analysis of RCTs comparing CA vs. AADs among patients with ventricular tachycardia and IHD. Our major findings indicate that CA significantly reduced appropriate ICD therapies and CV re-hospitalization, when compared to AADs, in patients with IHD while also being associated with a lower incidence of treatment-related adverse events. Additionally in an exploratory subgroup analysis, CA was superior to sotalol therapy when analysing a composite endpoint including appropriate ICD Shock, VT storm, and all-cause mortality (*Figure 3*).

Although moderate heterogeneity was observed for the primary endpoint, this was mainly driven by differences in trial design—particularly the prophylactic approach of SMASH-VT—and did not materially affect the direction or magnitude of the pooled effect.

Previous meta-analyses published on this topic evaluated VT ablation vs. medical therapy which is part of the VT management,^22^ or included patients with different structural heart disease.^23^ Of note, the present meta-analysis does not take into account VTs in the setting of electrical storms.^24^

This meta-analysis focuses on a head-to-head comparison of AADs vs. ablation. These data could guide physicians towards a tailored approach in cases of VT in patients with IHD. Indeed, patients with VT and acceptable life expectancy should be referred to CA considering all potential adverse events associated with long-term use of AADs. On the other side, in frail patients with shorter life expectancy, treatment with AADs, preferably amiodarone, is reasonable.^25^

In general, the results align with prior landmark studies and with recent guidelines. The 2022 European guidelines on SCD and VA positions VT ablation as class I indication in case of recurrent, symptomatic sustained monomorphic VT or ICD shocks for sustained monomorphic VT despite chronic amiodarone therapy in patients with IHD.^26,27^ The 2019 expert consensus statement also has VT ablation in class I in case of recurrent monomorphic VT despite chronic amiodarone or in case of any AAD is contraindicated or not tolerated.^28^ The main difference between these documents is that from 2019 expert consensus authors highlighted the potential benefit of VT ablation in case of AADs side effects. Both guidelines were written before the publication of two RCTs that have been included in the present meta-analysis.

The inclusion of the most recent RCTs (SURVIVE-VT and VANISH-2) in this meta-analysis provides an updated and clinically relevant evidence base.^20,21^ The consistent reduction in adverse events with CA across RCTs represents a novel and clinically meaningful finding. While procedural complications (e.g. perforation and bleeding) were observed, they were infrequent and outweighed by the higher rate of fatal and non-fatal drug toxicities, particularly with long-term amiodarone. As a matter of fact, the rate of long-term adverse events related to AAD range from 10% to 20% and it is associated mainly with liver, thyroid and lung disorders. The lower rate of adverse events with CA was largely driven by the higher long-term toxicity of amiodarone, while the comparison with sotalol showed no significant difference in safety. These data reinforce the concept that ablation, when performed in experienced centres, carries a more favourable long-term safety profile.^29^

The lack of mortality benefit of CA over AAD in this meta-analysis deserves careful interpretation and no real conclusion can be drawn, given that none of the included studies were powered for survival endpoints. Observational data suggest improved outcomes when VT is eliminated after ablation,^30^ but definitive survival benefit will require larger trials or pooled patient-level meta-analyses. While the effectiveness of AADs is relatively fixed, the outcomes of CA are likely to further improve with the ongoing optimization of imaging and substrate characterization. Future directions include not only the earlier use of ablation and integration of advanced high-density mapping and epicardial approaches, but also the enhanced integration of pre-procedural and intra-procedural imaging. The use of cardiac magnetic resonance imaging and computer tomography-based scar mapping, for example, is increasingly refining target identification and procedural planning.^31,32^ As recent studies illustrate, these evolving imaging-based strategies may enhance procedural precision, safety, and long-term efficacy.^33,34^ A better patient stratification based on these advanced scar imaging and substrate characteristics will also be critical for optimizing treatment strategies.^35–37^

An additional novelty in this meta-analysis is the subgroup analysis by AAD. Amiodarone, the most widely used AAD for VT in IHD, proved to have similar efficacy in terms of appropriate ICD shock reduction but is associated with several long-term side effects.^38^ On the other hand, Sotalol is frequently considered in patients with less severe ventricular dysfunction or intolerance to amiodarone.^39^ However, its anti-arrhythmic efficacy is modest when compared to amiodarone,^40^ and proarrhythmic risks^41^ including torsade de pointes, bradyarrhythmia, and heart failure limits its use in advanced ischaemic cardiomyopathy. The subgroup analysis showed that CA was associated with a significant reduction in the composite endpoint when compared with sotalol, suggesting that ablation provides superior arrhythmic control and safety in this setting. Therefore, our findings support prioritizing ablation over sotalol, particularly in high-risk ischaemic patients. Additionally, amiodarone should be preferably reserved only for acute management and patients should be informed about side effects.^42^

Overall, these findings support a strategy of early CA in ischaemic VT, particularly in patients with recurrent ICD therapies despite AADs, with ablation offering superior arrhythmic control and a more favourable safety profile.

Although mortality remains unaffected, ablation offers clear clinical and quality-of-life benefits and should be considered earlier in management algorithms, particularly in high-volume centres with proven expertise.

## Limitations

This analysis is limited by moderate heterogeneity in trial designs and control groups, and reliance on study-level rather than patient-level data. The meta-analysis was not prospectively registered (e.g. in PROSPERO), although it followed pre-defined selection and analysis criteria. Moreover, the high male prevalence (93%) across trials should be considered when interpreting these findings.

## Conclusion

In patients with IHD presenting with VT, CA significantly reduces appropriate ICD therapies and CV re-hospitalizations. Furthermore, CA is associated with a more favourable adverse event profile compared to AAD. The advantages of CA are particularly evident when compared with sotalol therapy. These findings support the early use of CA as a first-line or adjunctive strategy in appropriately selected patients with IHD.

## Supplementary Material

euaf302_Supplementary_Data

## Data Availability

The data that support the findings of this study are available on request from the corresponding author. The data are not publicly available due to privacy or ethical restrictions.
