# Supplementary material for: Catheter ablation vs. anti-arrhythmic drug therapy for ventricular tachycardia in ischaemic heart disease: a meta-analysis of randomized controlled trials
Source: Europace. 2025 Nov 29;27(12):euaf302. doi: 10.1093/europace/euaf302 (PMC12678166; doi:10.1093/europace/euaf302)
Supplement: euaf302_Supplementary_Data [file euaf302_supplementary_data.docx]

**Supplementary Appendix**

**Table of Contents**

- Figure S1. Forest plots for primary outcome and CV rehospitalization/adverse events 2
- Figure S2: Funnel plots for primary outcome 3
- Figure S3. Forest plots for secondary outcomes 4
- Figure S4. Subgroup analyses (ablation vs amiodarone / sotalol) in composite endpoint 5
- Table S1. AMSTAR 2 checklist compliance for the present meta-analysis 6
- Table S2. Risk of Bias (ROB 2) assessment for included randomized controlled trials 7


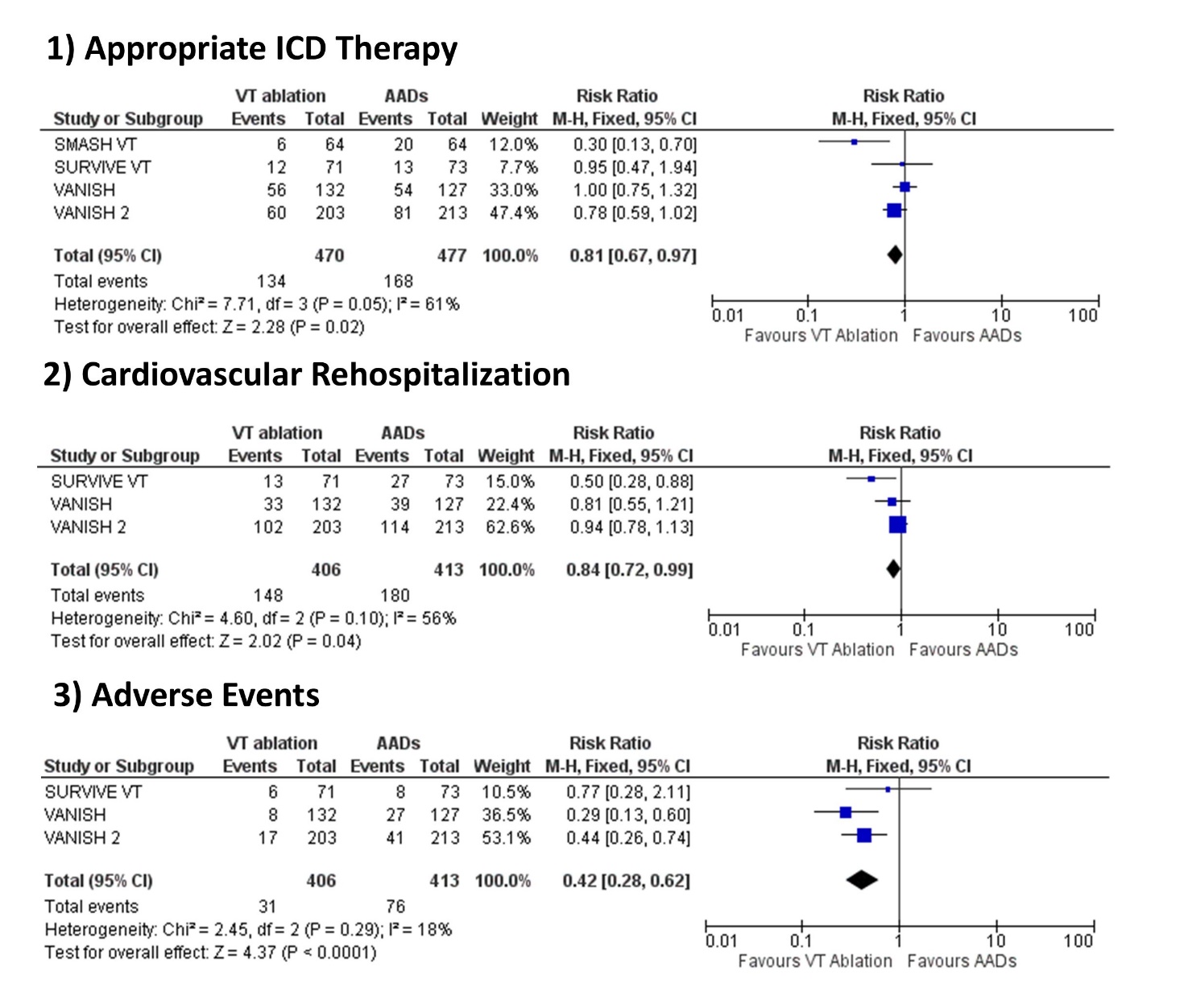


## **Figure S1** Forest plot comparing ventricular tachycardia (VT) ablation vs anti-arrhythmic drugs (AADs) for the primary outcome (Appropriate ICD therapy) and for Cardiovascular re-hospitalization and adverse events.


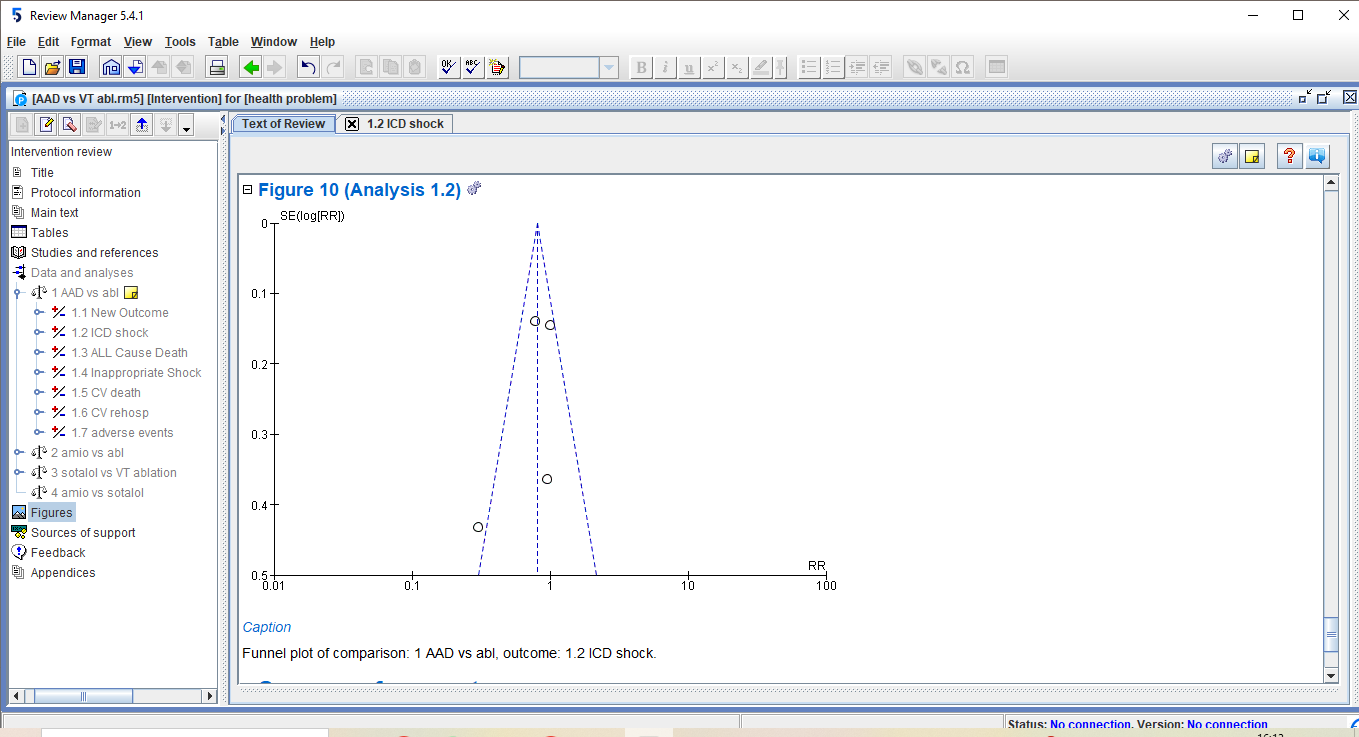


**Figure S2:** funnel plot of comparison of the primary end point (Appropriate ICD therapy) comparing ventricular tachycardia (VT) ablation vs anti-arrhythmic drugs.


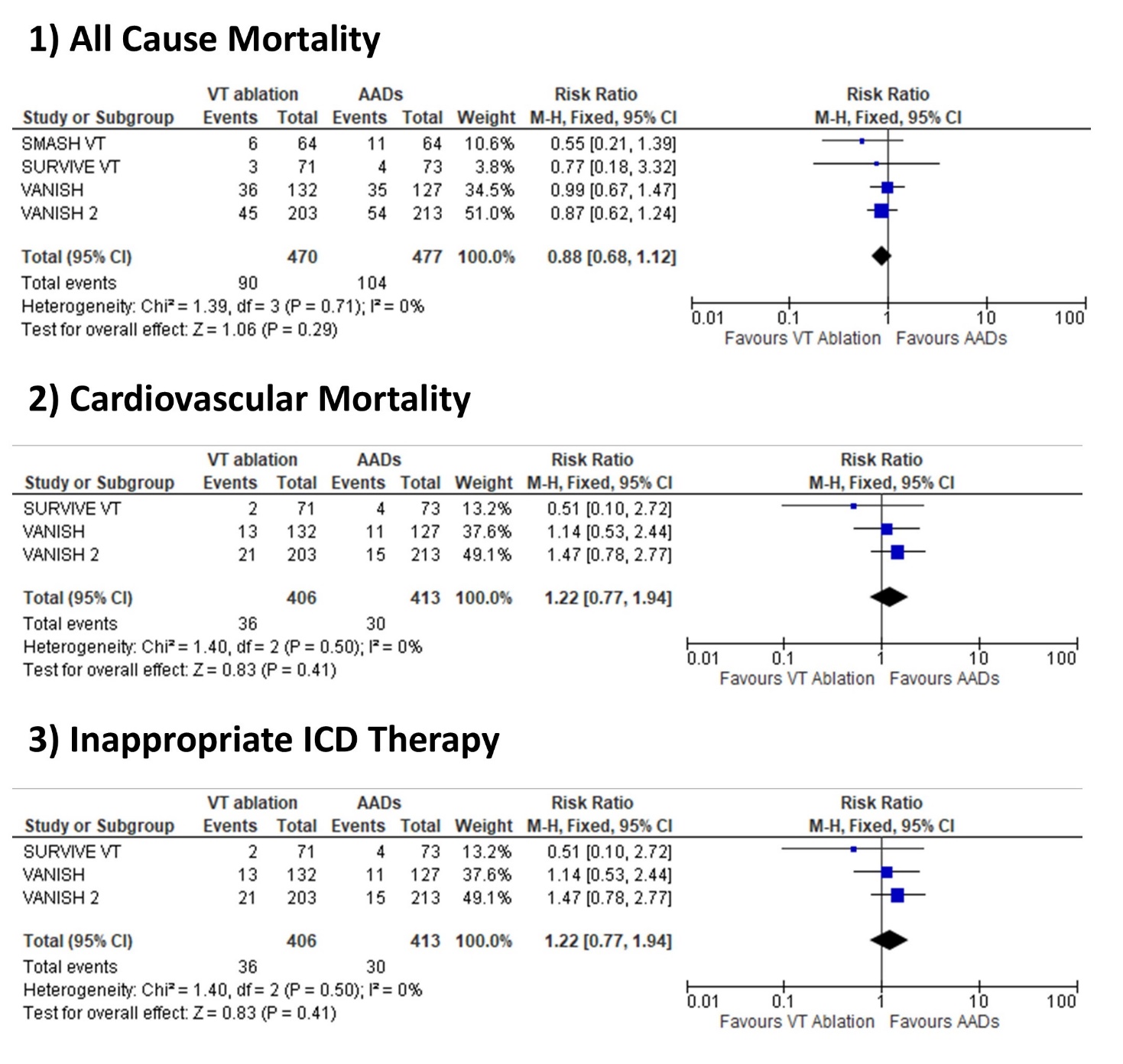


**Figure S3:** Forest plot comparing ventricular tachycardia (VT) ablation vs anti-arrhythmic drugs (AADs) for all-cause mortality (1), cardiovascular mortality (2) and inappropriate ICD therapy (3).


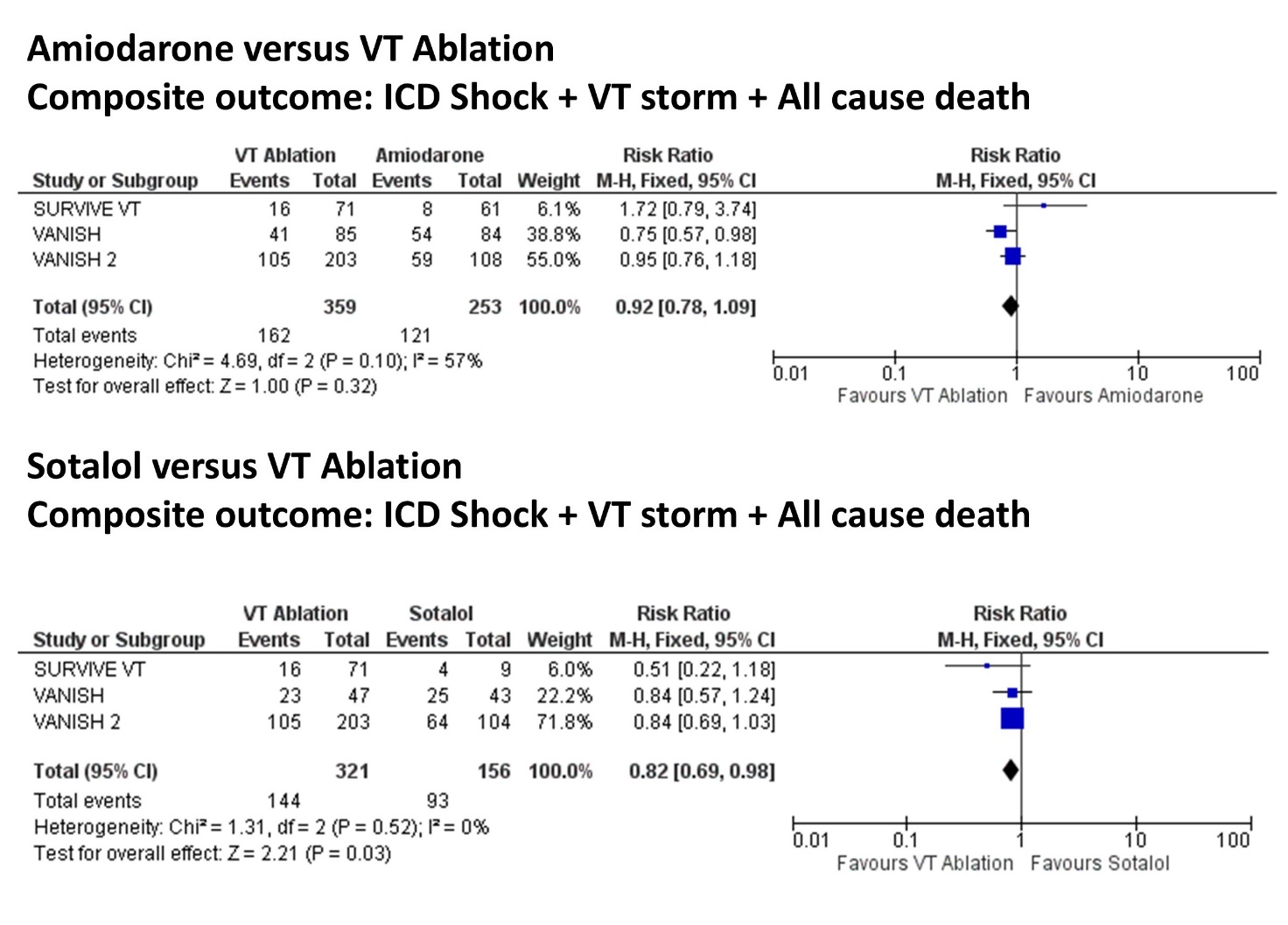


## **Figure S4.** Forest plot for composite endpoint (ICD shock, VT storm and all cause death) comparing

ventricular tachycardia (VT) ablation vs amiodarone or sotalol.

| **AMSTAR 2 Item** | **Description** | **Compliance in current meta-analysis** |
| --- | --- | --- |
| 1 | Research questions and inclusion criteria include PICO elements (Population, Intervention, Comparator, Outcomes). | ✓ Clearly defined in Introduction and Methods. |
| 2 | Review methods established prior to the conduct of the review (protocol registered). | ✓ The study followed a predefined protocol; no PROSPERO registration due to secondary nature of analysis (stated in Methods). |
| 3 | Explanation of study design inclusion (RCTs, observational, etc.). | ✓ Only randomized controlled trials (RCTs) were included, as specified in Methods. |
| 4 | Comprehensive literature search performed in at least two databases. | ✓ Search conducted in PubMed, Embase, and Cochrane databases up to July 2025. |
| 5 | Study selection performed in duplicate. | ✓ Two independent reviewers screened studies with adjudication by a third reviewer. |
| 6 | Data extraction performed in duplicate. | ✓ Data extracted independently by two reviewers using standardized forms. |
| 7 | List of included and excluded studies provided. | ✓ PRISMA flow diagram and study inclusion list reported in Supplementary Material. |
| 8 | Characteristics of included studies described in detail. | ✓ Study design, population, and outcomes summarized in Table 1. |
| 9 | Risk of bias assessment conducted for individual studies. | ✓ Performed using Cochrane ROB 2 tool. |
| 10 | Sources of funding for included studies reported. | ✓ Reported where available; none were industry-sponsored. |
| 11 | Appropriate meta-analytical methods used. | ✓ Random-effects model (DerSimonian–Laird) with pooled RRs and 95% CIs. |
| 12 | Potential impact of risk of bias discussed in interpretation. | ✓ Addressed in Discussion and sensitivity analyses. |
| 13 | Heterogeneity quantified and discussed. | ✓ I² values reported for all primary and secondary outcomes. |
| 14 | Publication bias assessed and addressed. | ✓ Evaluated via funnel plot (Supplementary Figure S2). |
| 15 | Adequate explanation of heterogeneity provided. | ✓ Explored via subgroup and sensitivity analyses (amiodarone vs sotalol). |
| 16 | Discussion reflects study quality and limitations. | ✓ Addressed in Discussion section. |
| 17 | Potential conflicts of interest declared. | ✓ Fully reported at the end of the manuscript. |

**Supplementary Table S1.** AMSTAR 2 checklist compliance for the present meta-analysis

| **Study (Year)** | **Randomization process** | **Deviations from intended interventions** | **Missing outcome data** | **Measurement of the outcome** | **Selection of the reported result** | **Overall risk of bias** | **Comments / Support for judgement** |
| --- | --- | --- | --- | --- | --- | --- | --- |
| **SMASH-VT (2007)** | Low | Some concerns | Low | Low | Low | Some concerns | Open-label; objective ICD-recorded endpoints; minor crossover allowed. |
| **VANISH (2016)** | Low | Some concerns | Low | Low | Low | Some concerns | Randomized; crossover permitted in AAD arm; objective outcome measurement. |
| **SURVIVE-VT (2022)** | Low | Low | Low | Low | Low | Low | Multicenter RCT with blinded outcome adjudication; minimal attrition. |
| **VANISH-2 (2025)** | Low | Some concerns | Low | Low | Low | Some concerns | Open-label; intention-to-treat design; objective device endpoints. |

**Supplementary Table S2**. Risk of Bias (ROB 2) assessment for included randomized controlled trials
